# Ultrasound Imaging Reveals Accelerated *In-utero* Development of a Sensory Apparatus in Echolocating Bats

**DOI:** 10.1038/s41598-019-41715-y

**Published:** 2019-03-27

**Authors:** Eran Amichai, Smadar Tal, Arjan Boonman, Yossi Yovel

**Affiliations:** 1 School of Zoology, Tel Aviv University, Tel Aviv-Yafo, Israel; 2 Sagol School of Neuroscience, Tel Aviv University, Tel Aviv-Yafo, Israel; 3 Koret School of Veterinary Medicine, The Hebrew University, Jerusalem, Israel

## Abstract

Organ development, both *in-utero* and after birth, follows a different path for every organ depending upon how early the newborn will use it. Perception of the environment using echolocation occurs very early in the life of neonatal bats. In nostril-emitting echolocating bats of the families Hipposideridae and Rhinolophidae, the shape and area of the nasal-horseshoe is crucial for echolocation emission. We therefore hypothesized that most of this organ’s ontogeny will be completed *in-utero* while skull and wings will develop slower and continue their growth after birth. We used intrauterine ultrasonography of pregnant females, and measured newborn *Asellia tridens* (Hipposideridae) to test our hypothesis at different stages of ontogeny. We found that horseshoe development is completed *in-utero* and neonates begin emitting precursor echolocation calls already two days after birth. In contrast, skull and forearm only develop to 70% and 40% of adult size (respectively), and continue development after birth.

## Introduction

Not all organs develop at the same rate during mammalian ontogeny, both *in-utero* and post-natal. Organs that are critical to the embryo’s or fetus’ survival, or are to be used immediately after birth, reach developmental completion (function, form and size) earlier than organs that are less critical or that will be used only later during ontogeny. In humans for example, the heart is the first functional organ, already beating by week four of gestation^1^, at which time the limbs are only beginning to form^2^. This chronology is observed in domestic dogs and cats as well^3^, though the absolute embryonic age in days is different. Bats, in contrast are born with fully developed hind limbs, enabling the neonate to hang onto its mother immediately after birth^4^. Sensory organs often depend on size for optimal functionality. For example, localization of the origin of a sound source in mammals depends on skull size for horizontal localization and on ear size for vertical localization^5^. Human babies are born with much smaller skulls and ears than adults and they remain smaller for a long period. Their brain must therefore constantly adapt to changes in sound interpretation, and for many months they exhibit poor sound localization performance until their skull and ears reach full size^6–9^. This sub-optimal performance is acceptable in altricial species, i.e. animals that are born in a relatively undeveloped stage and that are heavily dependent on parental care.

Precocial species, on the other hand, are self-reliant at an early stage and must therefore possess a fully-functional sensory system soon after birth. Old world horseshoe and leaf-nosed bats (Chiroptera: Rhinolophidae & Hipposideridae), though not precocial by definition, have a short dependent phase. Lesser horseshoe bats (*Rhinolophus hipposideros)*, for example, are able to fly within 18–20 days from birth^10^ and the trident leaf-nosed bat (*Asellia tridens*) is volant at three weeks^11^. This is especially rapid when considering the general longevity of bats^12^. These two bat families share several sensory characteristics: they produce constant-frequency (CF) echolocation signals which are emitted through the nostrils, and they rely on prey-induced Doppler shifts to detect prey^13,14^. Their need to detect minute frequency changes drove the evolution of the so-called ‘acoustic fovea’^15,16^ which is a dramatic over-representation of the bat’s specific emission frequency, in both the cochlea and brain. Moreover, bats in these two families have evolved special facial appendages which surround their nostrils and are essential for forming their echolocation beam and therefore crucial for spatial sensing^17–19^ (very good illustrations can be found in pp. 19 & 24 in Dietz 2007^20^). In order for it to function optimally, the size of the nose-leaf must be tightly correlated with the wavelength of the emitted signal^21^.

Bats of these families already echolocate (or produce precursor echolocation calls) within a few days after birth (see S1), their flight ability is indistinguishable from adults at around three weeks, and they hunt independently using echolocation by the age of *ca*. 6–7 weeks^11^. The newborns therefore must develop a fully functional echolocation system rapidly including a functional emitter and a proper signal design. When considering this rapid sensory development, we hypothesized that the nose-leaf apparatus development will be prioritized during pre-natal ontogeny, so that its size matches the adult emission frequency as fast as possible. We used B-mode ultrasound-imaging to measure horseshoe and skull sizes *in-utero* along the gestation in trident leaf-nosed bats (*Asellia tridens*). We found that indeed their emission related apparatus approaches an adult size prior to parturition, in contrast to skull width and forelimbs that lag behind.

## Results

### Differential organ development

**Figure 1:**
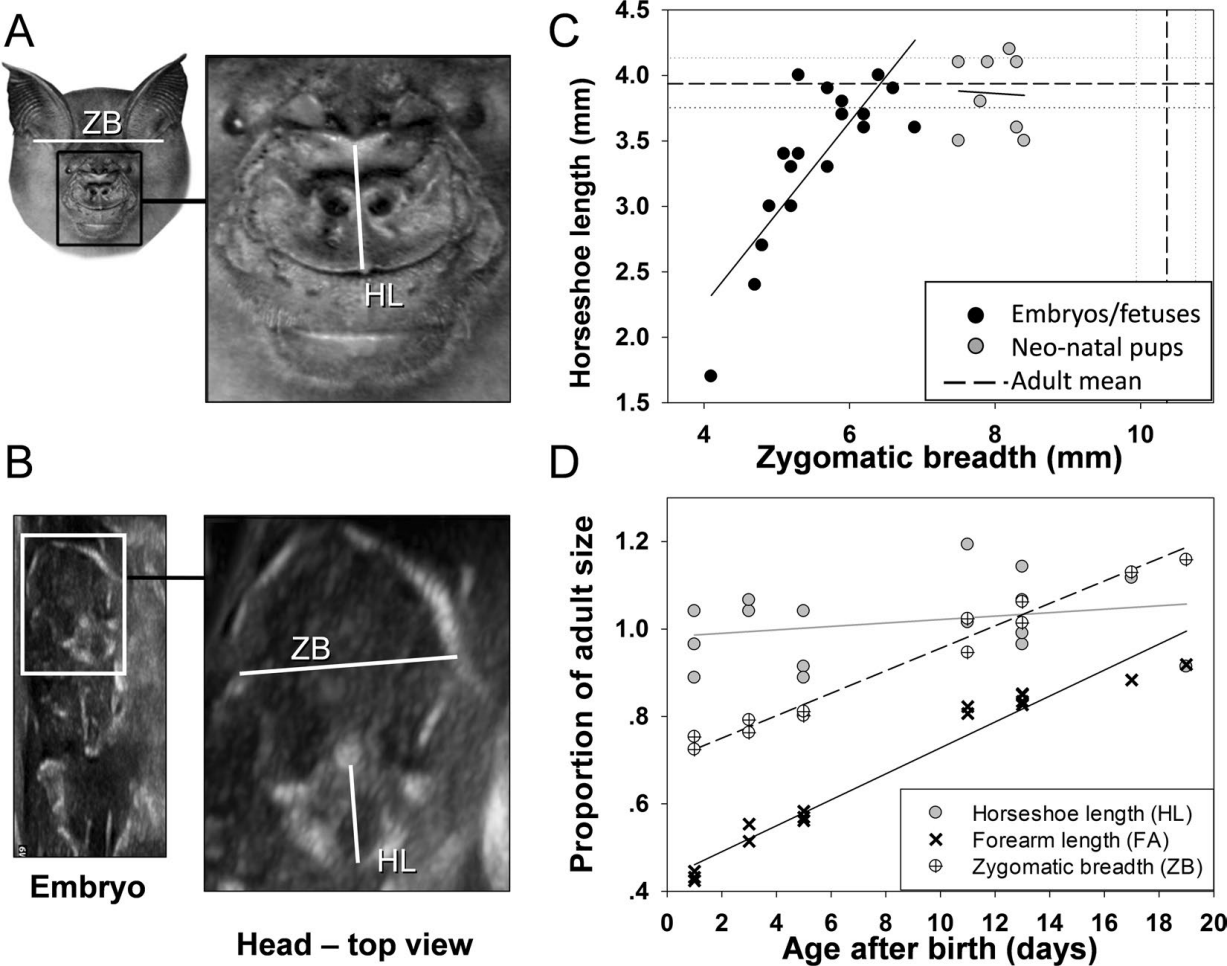
Differential organ development. Head morphometric parameters that were used in this study seen in an adult bat (**A**) and *in-utero* in an embryo/fetus (**B**): horseshoe length (HL), measured from the base of trident to the opposite edge of horseshoe, and zygomatic breadth (ZB), measured from the base of one zygomatic arch to the base of the other, forming the widest part of the skull. Embryonic and fetal development (**C**) of HL is much faster than ZB, reaching the adult size before birth while the ZB reaches only ~60% of adult size at the same time point (adult HL and ZB means are represented by horizontal and vertical dashed lines respectively, flanked by dotted lines at 2 standard errors). After birth (**D**) HL growth stops while ZB continues to grow, and forearm (FA) grows at a faster rate.

**Figure 2:**
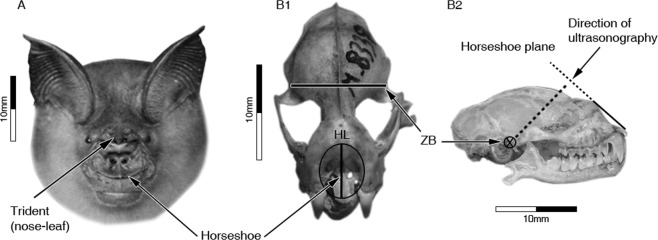
Morphometric parameters measurements and nomenclature. (**A**) The head and face of *A*. *tridens* showing the nasal emission apparatus including the nose-leaf (trident) and the horseshoe, the length of which was our metric HL – the two points we used to measure distance are depicted by arrows. (**B**) locations of measurements shown on *A*.*tridens* skull viewed from above. Zygomatic breadth (ZB) was measured across the skull from the root of one zygomatic arch to the root of the second. Also illustrated is the location of the horseshoe upon the skull (**B1**). Viewed from the side (**B2**) the right edge of the ZB is marked by X, located “behind” the horseshoe plane, perpendicular to the direction of ultrasonography.

*In-utero* development of the nose-leaf is faster than the skull’s growth rate. To quantify the horseshoe’s development, we measured the distance between the base of the trident and the opposite edge of the horseshoe (horseshoe length – HL, see Figs 1A,B, 2 and methods). This measurement serves as a good indicator for the total area of the horseshoe (see Methods). To quantify skull size, we measured the width of the skull at the root of the zygomatic arch, a metric referred to as zygomatic breadth, which has been shown to correlate with other skull dimensions^22^ and is often used in skull size indices (e.g.^23^) (ZB, Fig. 1B and methods Fig. 2B). As can be seen in Fig. 1, the horseshoe develops rapidly during gestation, completing its development *in-utero*. Horseshoe length (HL) reached adult dimensions before birth, while the skull developed slower and to a lesser extent, reaching less than 65% of the adult width ca. 2 weeks before parturition (Fig. 1C). An analysis of the nine largest fetuses (who are very likely the oldest ones) reveals that their HL did not differ significantly from the adults’ (3.86 ± 0.04 mm vs. 3.94 ± 0.08 mean ± SD, Two-sample t-test, t = −0.159, df = 29, p = 0.258). On the other hand, the mean ZB (6.3 ± 0.1 mm) only reached 61% of the adult mean (10.3 ± 0.2), t-test, t = −12.9, df = 29, p < 0.001). For further validation, we compared the horseshoe length of bats just before parturition to that of 1–5 days old pups and found no significant difference confirming that they reached the adult size *in utero* (compare the nine highest-value black points to the grey points in Fig. 1C: t-test, t = −0.748, df = 15, p = 0.466). To estimate when the skull reaches the adult size, we measured newborn pups and found that their skulls continued to grow at a similar pace (as in the womb) after birth until reaching the adult size around day 21 (Fig. 1D).

We compared post-birth horseshoe and skull development to that of the wing by measuring the forearm length (FA) of pups from birth to independent flight (day 21, Fig. 1D). While bats’ forearms are among the last organs to mature, and their growth rate varies across taxa^24^, within a species the growth curve is consistent among individuals and is a reliable metric to estimate pup age during post-natal ontogenesis^10,24^. While the horseshoe (at least the HL), does not grow appreciably during this period (the slope of a regression of the HL over time did not significantly differ from zero: *N* = 16, R = 0.32, t = 1.27, p = 0.22, 95% confidence intervals on the slope: lower = −0.01, upper = 0.05), both skull width and forearm length keep growing, with ZB increasing by ~30% and FA more than doubling within the three weeks after birth. The growth rate of the skull was ~0.25 mm/day while the rate of the forearm ~1.5 mm/day. The increase in FA length was significantly larger than that of the skull (an ANCOVA analysis of the increase with age of the two parameters reveals a significant difference between the slopes: F(1, 29) = 323.18, p < 0.0001). Despite its rapid growth, the forearm does not reach the adult size within 21 days, but only reaches ~92%.

### Echolocation ontogeny

Neonatal *A*. *tridens* start emitting precursor echolocation signals at a very early age – as early as two days after birth (Fig. S1). These signals are usually longer than adult signals, their constant frequency (CF) component is lower in comparison to the adults (105–109 kHz vs. ~120 kHz for the second harmonic (*f*2) which is the dominant one in this species), and they lack a pronounced frequency-modulated (FM) down-sweep at the end, which is typical for the adult signals. During post-birth ontogeny the signal’s design rapidly develops into the adult design: by day 12, the signal duration is already adult-like and the FM component is already pronounced. The emitted frequency at this stage is still lower than the adult ~110 kHz, and it remains lower than adult frequency when the pup begins to fly in the roost (112–115 kHz), but it reaches adult frequencies when the pup starts to leave the roost around day 21.

**Figure 3:**
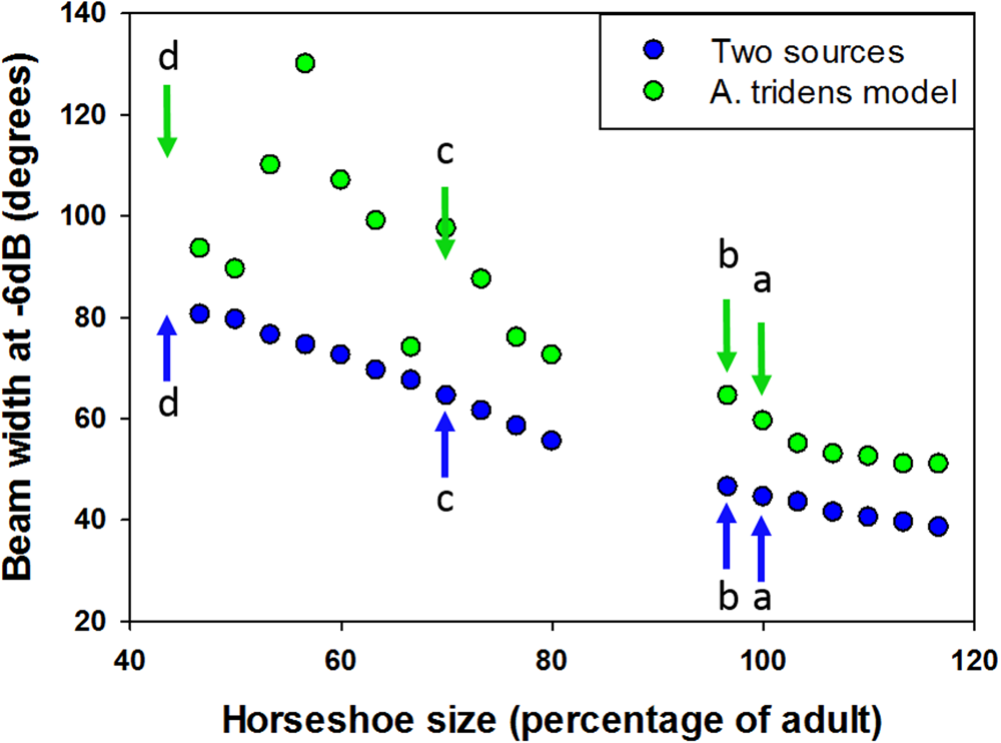
Echolocation beam width. Calculated beam widths at −6 dB intensity for emissions at 120 kHz. Beam width varies according to horseshoe size with 100 being actual adult *A*. *tridens* horseshoe size. Model I (blue dots) is a simple two coherent sound sources in air, separated by distance-between-nostrils. Model II (green dots) is the result of a computer simulation of sound emission through nostrils of a micro-CT scan of an adult bat. The arrows depict the adult predicted beam width with adult-size horseshoe and frequency (a), the predicted beam width for pups (adult-size horseshoe but lower frequency, (b)), the beam width that would have been generated if the pups were born with a horseshoe size proportional to their ZB (c), and the pup beam width that would have been generated if they were born with a horseshoe size proportional to FA (d).

As important as the echolocation signal’s design is, it is the beam which defines (with the ears) the animal’s sensory field of view^25–28^. In nostril-emitting bats, the beamform is largely determined by the ratio between the horseshoe-nose-leaf apparatus and the signal’s frequency. We estimated the bat’s horizontal beam-width (double sided 6 dB intensity drop) using two different models and we compared it to the width of the beam that would have been generated, had the bats not developed their nose-leaf *in-utero* as quickly as they do. We used an analytic model of two coherent sound sources (representing the nostrils) and a Finite Boundary Element simulation of a full 3D horseshoe and nose-leaf model (Methods). Based on both models, the new-born (double-sided) beam-width is 4–5 degrees wider than the adult (Fig. 3). This is a result of the slightly lower frequency of their echolocation signals. If, however, the pups were born with horseshoe dimensions proportionate to skull size (i.e. ~70% of adult size), the pup’s beam would have been 24° or 37° wider horizontally than that of the adults according to models I & II (respectively). The difference would be even larger had the pups been born with a horseshoe proportionate to forearm length: 40° or 57° wider than the adult beam (models I & II).

## Discussion

The rates at which organs develop *in-utero* and their prioritization differ according to each species’ ecological constraints and needs, and its evolutionary history. Here we show how echolocating bats’ reliance on a dominant sensory system – echolocation – leads to the rapid ontogeny of related organs, in this case: the horseshoe-leaf-nose apparatus for signal emission. The pup must develop a fully functional active sensing system within a short period of *ca*. 21 days when it begins to practice independent powered navigation. From this moment, it will have to develop independent hunting skills in a very short period (3–4 weeks), and hunting for small moving insects on the wing is a tough task.

In horseshoe and leaf-nosed bats, the size of the horseshoe is tightly related to the emitted beam’s width and directionality^18,19^. A beam too wide reduces localization abilities and it also means energy wasted – spreading in a much too wide sector. Our findings show that neonates start phonation at a very early age (Fig. S1). These echolocation calls are nasally emitted, unlike the mouth emitted isolation calls (see Fig. S2). The frequency of these early echolocation calls is lower than that of the adults, as has been documented in other bat species, and may be the result of the muscles controlling the vocal folds being immature, and not able to produce the tension required for the higher frequencies^29^. Alternatively, it maybe a result of shorter larynx, pharynx, rostrum, and supraglottal vocal tract, as those have not yet fully grown in the young bat^30^. While it may seem that lower frequency signals are highly detrimental due to the reliance on an acoustic fovea that matches adult frequencies, this is not necessarily the case. Just like the vocal tract, the cochlea undergoes postnatal development, and provides the feedback for vocalization maturation^31,32^. In contrast, the bat is born with a full-sized horseshoe, and the predicted emitted beam is only a few degrees wider than that of the adults (Fig. 3). Horseshoe size is not the only factor affecting beamforming. Musculature of the nostrils and their control, as well as the growth of the nasal passages affect emitted frequencies and thus beamforming in nasal-emitting bats^30,33^, as indeed our results show (see lower emitted frequencies in Fig. S1). The large size of the horseshoe at birth helps to generate a narrow echolocation beam despite the lower emission frequency of the new-born bats.

The pinnae seem to follow the same developmental path as the horseshoe – they seem to be disproportionately large at birth. Unfortunately, it was impossible to measure pinna size *in utero* as well as in young pups because during the first week, the ears are partly folded, (although at around four days of age the pup erects its ears when echolocating). Given the pinnae’s importance in estimating directionality and in determining the acoustic gaze^34^ it makes sense that they also reach the mature size very early.

Nasal-emitting bats thus provide a fascinating example of how evolutionary pressures combined with the animals’ life-history have rearranged the developmental trajectories of organs that are essential to their unique sensory system. We show how such processes can be studied non-invasively in wild animals.

## Methods

### Animals

Twenty-one adult pregnant female *Asellia tridens* bats were captured under permit from the Israel Nature and Parks Authority (2018/41843). Procedures were carried out in accordance to standards set by the Institutional Animal Care and Use Committee operating according to the Israel Health Ministry, ethics approval no. L-11-043. Researchers’ ethics approval numbers: EA: TAU-R 100493, ST: TAU-R 1011704, YY: TAU-R 100226. Bats were captured in their colonies and multiple ultrasound images were taken on site for later processing and measurement. Bats were released back into the roost after imaging, no longer than one hour after capture, in order to minimize stress. Eight post-partum females were captured so as to photograph their attached new pups. Nine older pups (ages 10–28 days; for age determination see below) were captured for growth rate measurements and echolocation ontogeny recordings.

### Ultrasound imaging and measurements

We used a portable ultrasound device (Mindray M9, Shenzhen Mindray Bio-medical Electronics, Co., China) with an L16-4HS hockey-stick 13 MHz transducer and B-mode imaging to visualize embryos and fetuses *in-utero* and measure their morphometric parameters. We applied ethanol to the bat’s stomach and gel to the probe. We then placed the probe on the abdomen of pregnant females and angled it to first identify fetal heartbeat and spine, and then find skull and nose-leaf. We chose an image angle that was perpendicular to both ZB and HL (see definitions in the next section) so it was possible to measure both ZB and HL from the same image (see Figs 1B and 2B). The effect of spherical aberration should be minor in comparison to other types of noise (such as placing the probe in the right angle) as the two lines are ~10 mm apart. Unlike with direct measurements, we could not be fully sure about imaging from the right angle. Therefore, several images were saved of each embryo and measurements were taken from the best 1–3 images (if more than one image was used, their values were averaged). The fact that the post-natal measurements of both ZB and HL were consistent with our *in-utero* measurements (creating a smooth continuum, Fig. 1C), suggests that we managed to measure the correct parameters. Each bat was handled for no longer than ten minutes.

It was not possible in all cases to find a suitable angle for measurements. This is a small bat (11–15 gr at the time of measurements), and the procedures were done without anesthesia and under time constraints. Individuals from which we were unable to obtain good images were omitted from the study. Unexpectedly, the more advanced the pregnancy was, the more difficult it was to obtain good images, since the large embryo was now fixed in its position and impossible to manipulate. In most cases the embryos were positioned with their wings folded between their own body and the mother’s spine, making it impossible to measure intrauterine forearm length.

### Morphometric measurements

We used two morphometric parameters to assess organ growth rates (see Figs 1A and 2B): 1. Zygomatic breadth (ZB) – the greatest width of the skull across at the distal roots of the zygomatic arches. 2. Horseshoe length (HL) – the distance between the base of the trident and the *inner* part of the horseshoe ridge at the line between (and perpendicular to) the nostrils. This metric serves as a proxy for horseshoe area. To ascertain this, we measured horseshoe length and horseshoe width at the widest point (HW) of 21 adult individuals and tested the two metrics for correlation. The results showed a significant positive correlation (Pearson correlation, N = 21, correlation coefficient = 0.6, p = 0.004). HW is itself positively correlated with the distance between the nostrils (correlation coefficient = 0.7, p < 0.001). While one must be careful when comparing growth rates of hard versus soft tissue organs, we do this here to highlight these differences.

For adults and neonatal pups, ZB was measured directly using a caliper (accuracy 0.02 mm, Mitutoyo, Japan). HL was measured both using caliper and from top-view photographs (normalized to a scale) to confirm accuracy. Measurements from photographs were used for analysis. For *in-utero* measurements, all parameters were derived from the ultrasound images (see next section).

To characterize post-natal growth, we used forearm length measurements (FA) in addition to the parameters described above. This was unfortunately impossible to measure *in-utero* (see above).

To verify that our *in-utero* measurements of HL were comparable to post-natal measurements, we placed a preserved specimen from a museum collection (The Steinhardt Museum of Natural History, Israel) in a water-filled latex balloon and measured its HL using ultrasound imaging. In this validation, both the Ultrasound and the external photos (the direct measurements) were acquired exactly like in the experiment itself. We then removed the specimen and measured its HL directly. The procedure was repeated with four specimens. The two measurements differed by 0.28 ± 0.15 mm (mean ± se).

It was very difficult to recognize the forearm of the developing bat *in-utero* and therefore we did not use it.

### Echolocation

All echolocation signals were recorded using a hand-held full-spectrum recorder (D1000, Pettersson, Sweden) with a sampling rate of 384 kHz. We recorded echolocating hand-held bats from a distance of one meter. Signals were analyzed using SASLab Pro software (Avisoft Bioacoustics, Germany). Signal durations were measured from the waveform (±9db from peak). Frequencies were measured from the spectrograms (FFT length: 256, overlap: 87.5%).

### Pup age determination

Post-natal pup age was determined using the ratio of adult-to-pup forearm length: Each pup’s forearm length (FA) was measured and its percentage of adult mean FA length was calculated. We then compared this to values in a known growth curve of *Rhinolophus hipposideros*^10^. Though these two species belong to different families, they have an almost identical pregnancy length, parturition time, time to independent flight, and ratio of adult-to-pup FA length at birth and at first flight^10,11^. Moreover, while FA growth rate may vary between taxa, within species growth curves are consistent^24^, and can therefore be used to reliably estimate pup age during the post-birth ontogenesis period.

### Beam width modeling

To investigate beamforming and its relation with nose-leaf dimensions we used two models: model I was a simple two coherent sound sources in air model (e.g.^35^). The sources were separated by a distance representing the distance between the nostrils of the bat. We analytically calculated the theoretical beam at 50 cm distance based on the phase difference between the two coherent sound sources emanating from the nostrils. The relative amplitude was calculated on the basis of the sum of the phases at every angle.

Model II used a Finite Boundary Element simulation of a full 3D nose-leaf model - a computer-generated simulation of echolocation calls emitted through the nostrils of a detailed adult *Asellia tridens* head. We first scanned the head of a freshly dead adult bat in a micro CT scanner (with a voxel size of 38.9 µm^3^, TomoScope, Synergy Twin). The generated dicom slices were merged into stl format by using Amira 6.2.0 3D visualization software (https://www.fei.com/software/amira-3d-for-life-sciences/), using adaptive thresholding for each region. The model head was imported into Meshlab (http://www.meshlab.net/) in which all faces were reoriented coherently, making sure that all normals were oriented into “air” and checking genus, absence of holes and two-manifoldness.

Finally, the model head object was imported into Matlab to carry out the acoustic computations using the Matlab-based boundary element model (‘Bemfa’, www.ee.bgu.ac.il/tourbabv/bemfa). With Bemfa, we measured the steady state complex sound pressure level emanating from the head at 50 cm distance with a 1 Pa (1 N/m^2) sound source placed inside the bat’s sound chamber. We assessed the sound pressure radially at 50 cm distance at points separated by 5 degrees in the hemisphere in front of the bat. We ran the simulation for different frequencies, leading to the range of beam-width-nose-leaf size relationships as presented in this paper.

For a more detailed description of the methods used to create model II, please refer to Vanderelst *et al*.^36^, which we followed.

## Supplementary information

Supplementary figures
