# Supplementary material for: Ultrasound Imaging Reveals Accelerated In-utero Development of a Sensory Apparatus in Echolocating Bats
Source: Sci Rep. 2019 Mar 27;9:5275. doi: 10.1038/s41598-019-41715-y (PMC6437157; doi:10.1038/s41598-019-41715-y)
Supplement: Supplementary file 1 — Supplementary figures [file 41598_2019_41715_MOESM1_ESM.pdf]

Ultrasound Imaging Reveals Accelerated *In-utero* Development of Sensory Organs in Echolocating Bats

Eran Amichai[1*], Smadar Tal[1], Arjan Boonman[1], Yossi Yovel[1,2*]

[1] School of Zoology, Tel Aviv University

[2] Sagol School of Neuroscience, Tel Aviv University

[*] Correspondence to: eranamichai@gmail.com

Supplementary materials including figures S1, & S2

**Age (days)**	***N* (ind. X signals/ind.)**	**Spectrogram**	**CF Frequency (kHz) of *f*2**	**Flight ability**
2	2 X 20	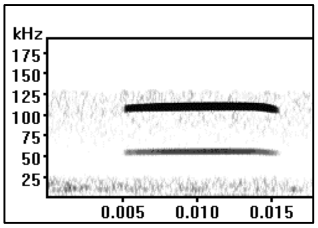	105-109	Non-volant: on mother, no wing movements
10	3 X 20	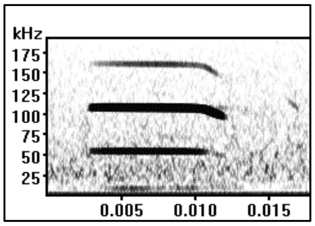	105-110	Pre-volant: on mother, wing flexing.
12	2 X 20	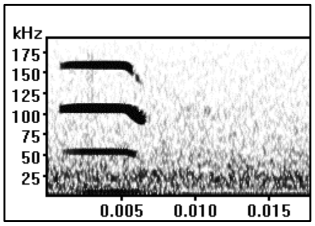	106-111	Pre-volant: on mother, wing flexing.
16	1 X 30	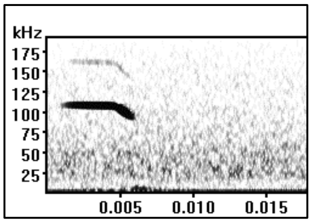	108-110	Pre-volant: on and off mother, short flights in roost, altitude not maintained long.
18	1 X 30	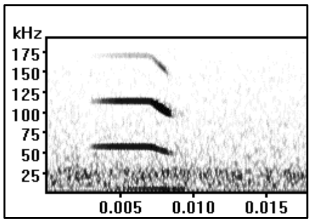	112-115	Volant: on and off mother, complex, sustained flight in roost only.
28	3 X 20	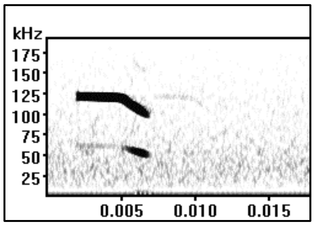	118-121	Volant: off mother, independent flights including foraging outside roost.

Figure S1: Ontogeny of echolocation signal design. Initial echolocation pulses are different in spectro-temporal parameters from adults' pulses. As the pup grows the signal parameters approach adult values. The fundamental harmonic which is typically suppressed in these bats is present in most cases, probably due to the bats mouths being open (protest vocalizations for being held in hand) during the recordings.

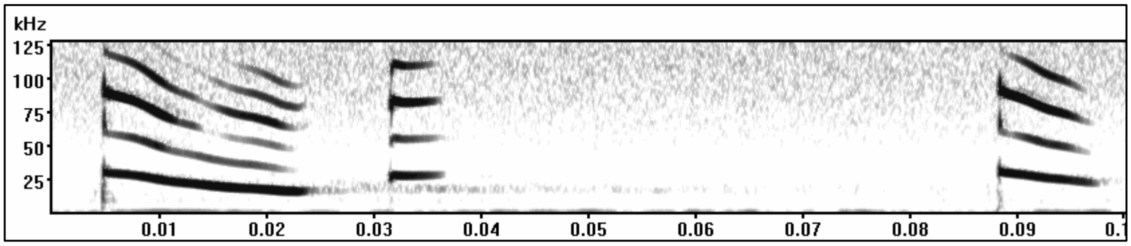

Figure S2: Neo-natal isolation calls. Spectrogram of a 2 days old pup’s isolation call. These calls are emitted from the mouth, and unlike echolocation calls (even at this age) they do not maintain a constant frequency, and contain more harmonics.
